# A scoping review of the literature featuring research ethics and research integrity cases

**DOI:** 10.1186/s12910-021-00620-8

**Published:** 2021-04-30

**Authors:** Anna Catharina Vieira Armond, Bert Gordijn, Jonathan Lewis, Mohammad Hosseini, János Kristóf Bodnár, Soren Holm, Péter Kakuk

**Affiliations:** 1 Department of Behavioural Sciences, Faculty of Medicine, University of Debrecen, Móricz Zsigmond krt. 22. III. Apartman Diákszálló, Debrecen, 4032 Hungary; 2 Institute of Ethics, School of Theology, Philosophy and Music, Dublin City University, Dublin, Ireland; 3 Centre for Social Ethics and Policy, School of Law, University of Manchester, Manchester, UK; 4 Center for Medical Ethics, HELSAM, Faculty of Medicine, University of Oslo, Oslo, Norway; 5 Center for Ethics and Law in Biomedicine, Central European University, Budapest, Hungary

**Keywords:** Research ethics, Research integrity, Scientific misconduct, Cases, Review

## Abstract

**Background:**

The areas of Research Ethics (RE) and Research Integrity (RI) are rapidly evolving. Cases of research misconduct, other transgressions related to RE and RI, and forms of ethically questionable behaviors have been frequently published. The objective of this scoping review was to collect RE and RI cases, analyze their main characteristics, and discuss how these cases are represented in the scientific literature.

**Methods:**

The search included cases involving a violation of, or misbehavior, poor judgment, or detrimental research practice in relation to a normative framework. A search was conducted in PubMed, Web of Science, SCOPUS, JSTOR, Ovid, and Science Direct in March 2018, without language or date restriction. Data relating to the articles and the cases were extracted from case descriptions.

**Results:**

A total of 14,719 records were identified, and 388 items were included in the qualitative synthesis. The papers contained 500 case descriptions. After applying the eligibility criteria, 238 cases were included in the analysis. In the case analysis, fabrication and falsification were the most frequently tagged violations (44.9%). The non-adherence to pertinent laws and regulations, such as lack of informed consent and REC approval, was the second most frequently tagged violation (15.7%), followed by patient safety issues (11.1%) and plagiarism (6.9%). 80.8% of cases were from the Medical and Health Sciences, 11.5% from the Natural Sciences, 4.3% from Social Sciences, 2.1% from Engineering and Technology, and 1.3% from Humanities. Paper retraction was the most prevalent sanction (45.4%), followed by exclusion from funding applications (35.5%).

**Conclusions:**

Case descriptions found in academic journals are dominated by discussions regarding prominent cases and are mainly published in the news section of journals. Our results show that there is an overrepresentation of biomedical research cases over other scientific fields compared to its proportion in scientific publications. The cases mostly involve fabrication, falsification, and patient safety issues. This finding could have a significant impact on the academic representation of misbehaviors. The predominance of fabrication and falsification cases might diverge the attention of the academic community from relevant but less visible violations, and from recently emerging forms of misbehaviors.

**Supplementary Information:**

The online version contains supplementary material available at 10.1186/s12910-021-00620-8.

## Background

There has been an increase in academic interest in research ethics (RE) and research integrity (RI) over the past decade. This is due, among other reasons, to the changing research environment with new and complex technologies, increased pressure to publish, greater competition in grant applications, increased university-industry collaborative programs, and growth in international collaborations [1]. In addition, part of the academic interest in RE and RI is due to highly publicized cases of misconduct [2].

There is a growing body of published RE and RI cases, which may contribute to public attitudes regarding both science and scientists [3]. Different approaches have been used in order to analyze RE and RI cases. Studies focusing on ORI files (Office of Research Integrity) [2], retracted papers [4], quantitative surveys [5], data audits [6], and media coverage [3] have been conducted to understand the context, causes, and consequences of these cases.

Analyses of RE and RI cases often influence policies on responsible conduct of research [1]. Moreover, details about cases facilitate a broader understanding of issues related to RE and RI and can drive interventions to address them. Currently, there are no comprehensive studies that have collected and evaluated the RE and RI cases available in the academic literature. This review has been developed by members of the EnTIRE consortium to generate information on the cases that will be made available on the Embassy of Good Science platform (www.embassy.science). Two separate analyses have been conducted. The first analysis uses identified research articles to explore how the literature presents cases of RE and RI, in relation to the year of publication, country, article genre, and violation involved. The second analysis uses the cases extracted from the literature in order to characterize the cases and analyze them concerning the violations involved, sanctions, and field of science.

## Methods

This scoping review was performed according to the Preferred Reporting Items for Systematic Reviews and Meta-Analyses (PRISMA) statement and PRISMA Extension for Scoping Reviews (PRISMA-ScR). The full protocol was pre-registered and it is available at https://ec.europa.eu/research/participants/documents/downloadPublic?documentIds=080166e5bde92120&appId=PPGMS.

### Eligibility

Articles with non-fictional case(s) involving a violation of, or misbehavior, poor judgment, or detrimental research practice in relation to a normative framework, were included. Cases unrelated to scientific activities, research institutions, academic or industrial research and publication were excluded. Articles that did not contain a substantial description of the case were also excluded.

A normative framework consists of explicit rules, formulated in laws, regulations, codes, and guidelines, as well as implicit rules, which structure local research practices and influence the application of explicitly formulated rules. Therefore, if a case involves a violation of, or misbehavior, poor judgment, or detrimental research practice in relation to a normative framework, then it does so on the basis of explicit and/or implicit rules governing RE and RI practice.

### Search strategy

A search was conducted in PubMed, Web of Science, SCOPUS, JSTOR, Ovid, and Science Direct in March 2018, without any language or date restrictions. Two parallel searches were performed with two sets of medical subject heading (MeSH) terms, one for RE and another for RI. The parallel searches generated two sets of data thereby enabling us to analyze and further investigate the overlaps in, differences in, and evolution of, the representation of RE and RI cases in the academic literature. The terms used in the first search were: (("research ethics") AND (violation OR unethical OR misconduct)). The terms used in the parallel search were: (("research integrity") AND (violation OR unethical OR misconduct)). The search strategy’s validity was tested in a pilot search, in which different keyword combinations and search strings were used, and the abstracts of the first hundred hits in each database were read (Additional file 1).

After searching the databases with these two search strings, the titles and abstracts of extracted items were read by three contributors independently (ACVA, PK, and KB). Articles that could potentially meet the inclusion criteria were identified. After independent reading, the three contributors compared their results to determine which studies were to be included in the next stage. In case of a disagreement, items were reassessed in order to reach a consensus. Subsequently, qualified items were read in full.

### Data extraction

Data extraction processes were divided by three assessors (ACVA, PK and KB). Each list of extracted data generated by one assessor was cross-checked by the other two. In case of any inconsistencies, the case was reassessed to reach a consensus. The following categories were employed to analyze the data of each extracted item (where available): (I) author(s); (II) title; (III) year of publication; (IV) country (according to the first author's affiliation); (V) article genre; (VI) year of the case; (VII) country in which the case took place; (VIII) institution(s) and person(s) involved; (IX) field of science (FOS-OECD classification)[7]; (X) types of violation (see below); (XI) case description; and (XII) consequences for persons or institutions involved in the case.

Two sets of data were created after the data extraction process. One set was used for the analysis of articles and their representation in the literature, and the other set was created for the analysis of cases. In the set for the analysis of articles, all eligible items, including duplicate cases (cases found in more than one paper, e.g. Hwang case, Baltimore case) were included. The aim was to understand the historical aspects of violations reported in the literature as well as the paper genre in which cases are described and discussed. For this set, the variables of the year of publication (III); country (IV); article genre (V); and types of violation (X) were analyzed.

For the analysis of cases, all duplicated cases and cases that did not contain enough information about particularities to differentiate them from others (e.g. names of the people or institutions involved, country, date) were excluded. In this set, prominent cases (i.e. those found in more than one paper) were listed only once, generating a set containing solely unique cases. These additional exclusion criteria were applied to avoid multiple representations of cases. For the analysis of cases, the variables: (VI) year of the case; (VII) country in which the case took place; (VIII) institution(s) and person(s) involved; (IX) field of science (FOS-OECD classification); (X) types of violation; (XI) case details; and (XII) consequences for persons or institutions involved in the case were considered.

### Article genre classification

We used ten categories to capture the differences in genre. We included a case description in a “news” genre if a case was published in the news section of a scientific journal or newspaper. Although we have not developed a search strategy for newspaper articles, some of them (e.g. New York Times) are indexed in scientific databases such as Pubmed. The same method was used to allocate case descriptions to “editorial”, “commentary”, “misconduct notice”, “retraction notice”, “review”, “letter” or “book review”. We applied the “case analysis” genre if a case description included a normative analysis of the case. The “educational” genre was used when a case description was incorporated to illustrate RE and RI guidelines or institutional policies.

### Categorization of violations

For the extraction process, we used the articles’ own terminology when describing violations/ethical issues involved in the event (e.g. plagiarism, falsification, ghost authorship, conflict of interest, etc.) to tag each article. In case the terminology was incompatible with the case description, other categories were added to the original terminology for the same case. Subsequently, the resulting list of terms was standardized using the list of major and minor misbehaviors developed by Bouter and colleagues [8]. This list consists of 60 items classified into four categories: Study design, data collection, reporting, and collaboration issues. (Additional file 2).

## Results

### Systematic search

**Fig. 1:**
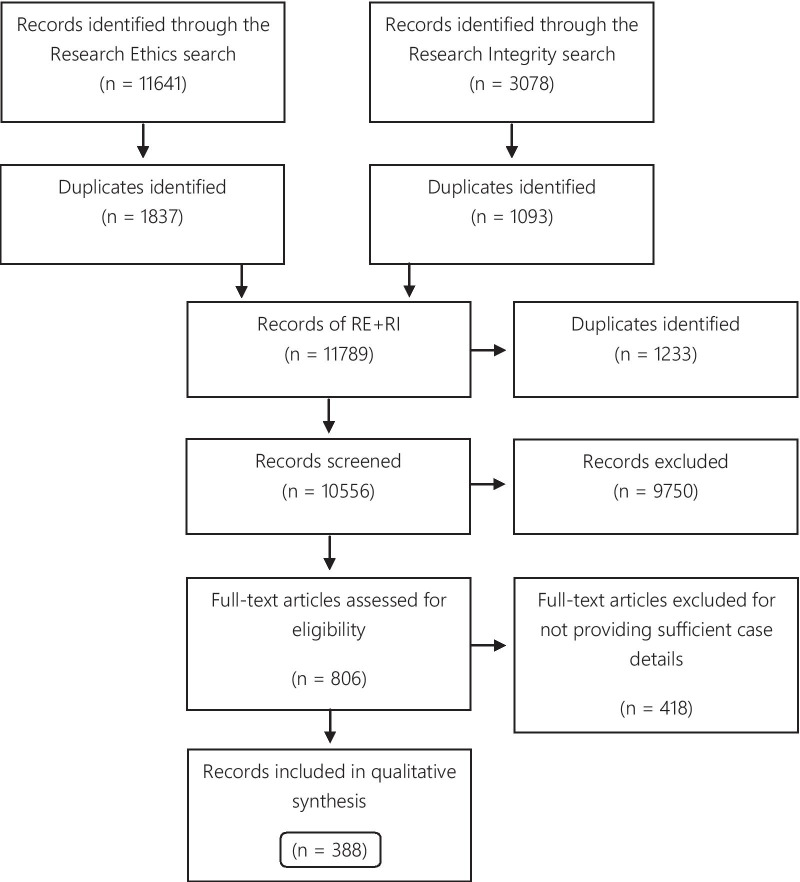
Flow diagram

A total of 11,641 records were identified through the RE search and 3078 in the RI search. The results of the parallel searches were combined and the duplicates removed. The remaining 10,556 records were screened, and at this stage, 9750 items were excluded because they did not fulfill the inclusion criteria. 806 items were selected for full-text reading. Subsequently, 388 articles were included in the qualitative synthesis (Fig. 1).

Of the 388 articles, 157 were only identified via the RE search, 87 exclusively via the RI search, and 144 were identified via both search strategies. The eligible articles contained 500 case descriptions, which were used for the analysis of the publications articles analysis. 256 case descriptions discussed the same 50 cases. The Hwang case was the most frequently described case, discussed in 27 articles. Furthermore, the top 10 most described cases were found in 132 articles (Table 1).

**Table 1:** Top 10 most described cases

Cases	Articles	Date range
1. Hwang	27	2005–2016
2. Baltimore/Imanishi-kari	24	1990–2007
3. Gallo	21	1990–2010
4. Fisher/Poisson	12	1994–1997
5. Schön	10	2002–2014
6. Luk Van Parijs	9	1998–2011
7. Poehlman	8	2005–2010
8. Boldt	8	2011–2014
9. Wakefield	7	2004–2013
10. CNEP	6	2006–2010

For the analysis of cases, 206 (41.2% of the case descriptions) duplicates were excluded, and 56 (11.2%) cases were excluded for not providing enough information to distinguish them from other cases, resulting in 238 eligible cases.

#### Violations

##### Analysis of the articles

**Fig. 2:**
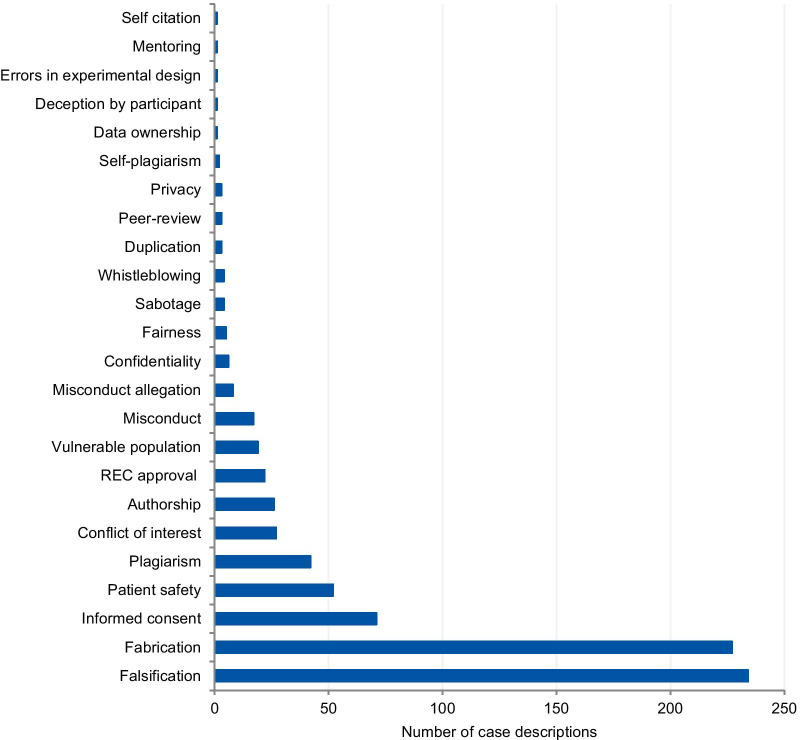
Tagged violations from the article analysis

The categories used to classify the violations include those that pertain to the different kinds of scientific misconduct (falsification, fabrication, plagiarism), detrimental research practices (authorship issues, duplication, peer-review, errors in experimental design, and mentoring), and “other misconduct” (according to the definitions from the National Academies of Sciences and Medicine, [1]). Each case could involve more than one type of violation. The majority of cases presented more than one violation or ethical issue, with a mean of 1.56 violations per case. Figure 2 presents the frequency of each violation tagged to the articles. Falsification and fabrication were the most frequently tagged violations. The violations accounted respectively for 29.1% and 30.0% of the number of taggings (n = 780), and they were involved in 46.8% and 45.4% of the articles (n = 500 case descriptions). Problems with informed consent represented 9.1% of the number of taggings and 14% of the articles, followed by patient safety (6.7% and 10.4%) and plagiarism (5.4% and 8.4%). Detrimental research practices, such as authorship issues, duplication, peer-review, errors in experimental design, mentoring, and self-citation were mentioned cumulatively in 7.0% of the articles.

##### Analysis of the cases

**Fig. 3:**
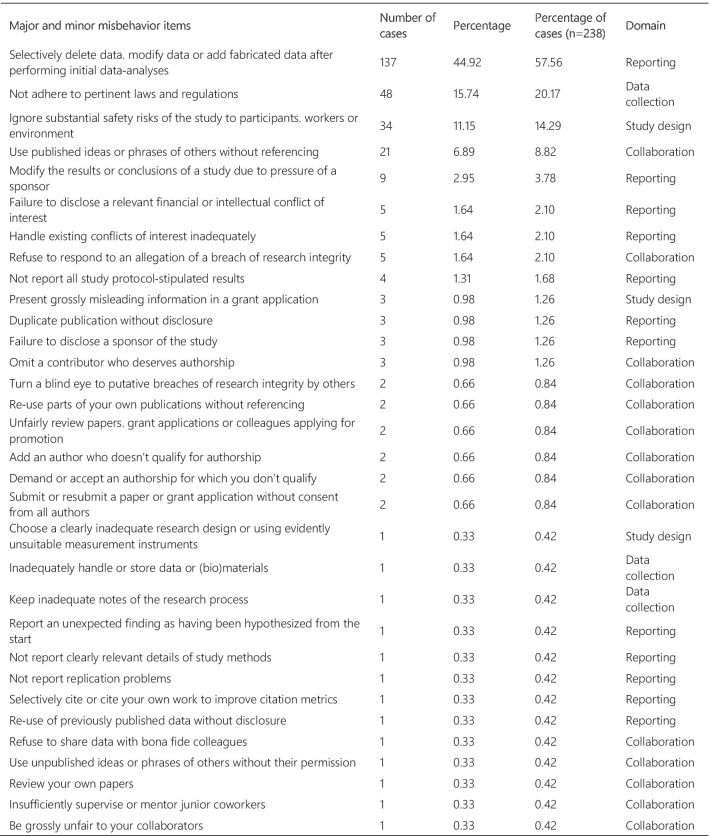
Major and minor misbehavior items from the analysis of cases

Figure 3 presents the frequency and percentage of each violation found in the cases. Each case could include more than one item from the list. The 238 cases were tagged 305 times, with a mean of 1.28 items per case. Fabrication and falsification were the most frequently tagged violations (44.9%), involved in 57.7% of the cases (n = 238). The non-adherence to pertinent laws and regulations, such as lack of informed consent and REC approval, was the second most frequently tagged violation (15.7%) and involved in 20.2% of the cases. Patient safety issues were the third most frequently tagged violations (11.1%), involved in 14.3% of the cases, followed by plagiarism (6.9% and 8.8%). The list of major and minor misbehaviors [8] classifies the items into study design, data collection, reporting, and collaboration issues. Our results show that 56.0% of the tagged violations involved issues in reporting, 16.4% in data collection, 15.1% involved collaboration issues, and 12.5% in the study design. The items in the original list that were not listed in the results were not involved in any case collected.

#### Article genre

**Fig. 4:**
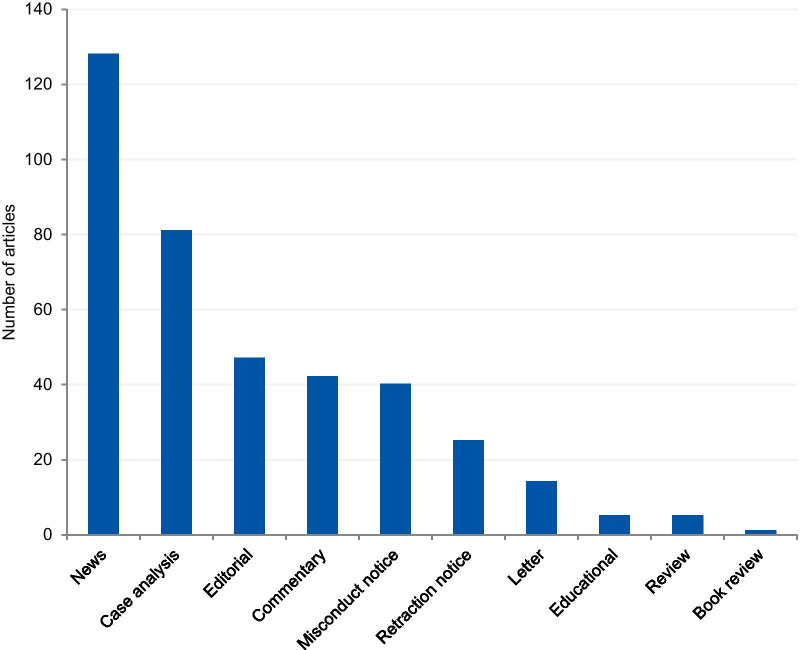
Article genre of included articles

The articles were mostly classified into “news” (33.0%), followed by “case analysis” (20.9%), “editorial” (12.1%), “commentary” (10.8%), “misconduct notice” (10.3%), “retraction notice” (6.4%), “letter” (3.6%), “educational paper” (1.3%), “review” (1%), and “book review” (0.3%) (Fig. 4). The articles classified into “news” and “case analysis” included predominantly prominent cases. Items classified into “news” often explored all the investigation findings step by step for the associated cases as the case progressed through investigations, and this might explain its high prevalence. The case analyses included mainly normative assessments of prominent cases. The misconduct and retraction notices included the largest number of unique cases, although a relatively large portion of the retraction and misconduct records could not be included because of insufficient case details. The articles classified into “editorial”, “commentary” and “letter” also included unique cases.

#### Date

##### Article analysis

**Fig. 5:**
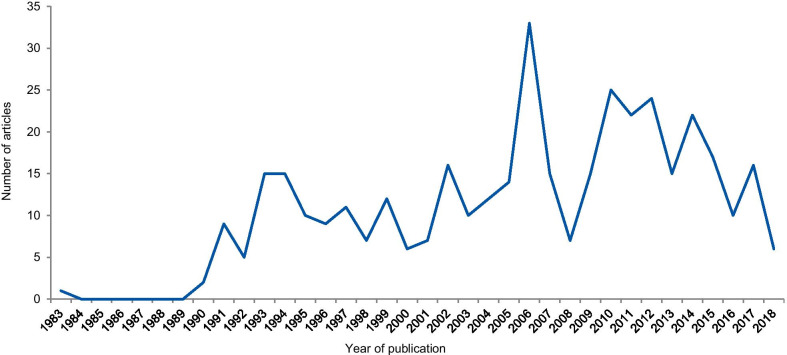
Frequency of articles according to the year of publication

The dates of the eligible articles range from 1983 to 2018 with notable peaks between 1990 and 1996, most probably associated with the Gallo [9] and Imanishi-Kari cases [10], and around 2005 with the Hwang [11], Wakefield [12], and CNEP trial cases [13] (Fig. 5). The trend line shows an increase in the number of articles over the years.

##### Case analysis

**Fig. 6:**
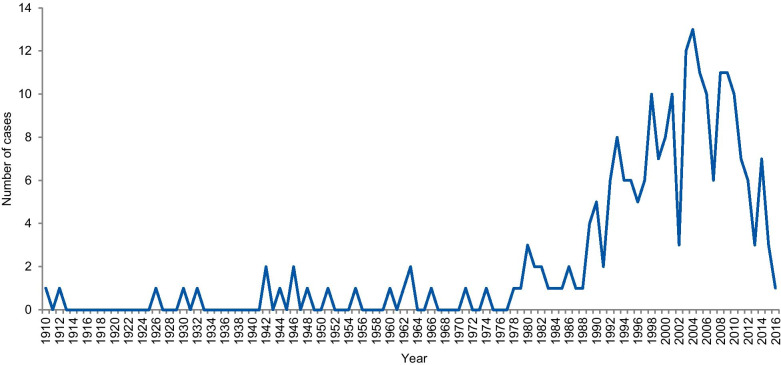
Frequency of cases per year

The dates of included cases range from 1798 to 2016. Two cases occurred before 1910, one in 1798 and the other in 1845. Figure 6 shows the number of cases per year from 1910. An increase in the curve started in the early 1980s, reaching the highest frequency in 2004 with 13 cases.

#### Geographical distribution

##### Article analysis

**Fig. 7:**
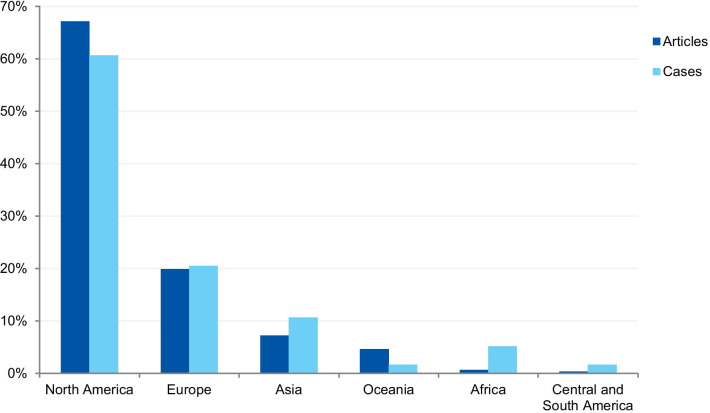
Percentage of articles and cases by continent

The first analysis concerned the authors’ affiliation and the corresponding author’s address. Where the article contained more than one country in the affiliation list, only the first author’s location was considered. Eighty-one articles were excluded because the authors’ affiliations were not available, and 307 articles were included in the analysis. The articles originated from 26 different countries (Additional file 3). Most of the articles emanated from the USA and the UK (61.9% and 14.3% of articles, respectively), followed by Canada (4.9%), Australia (3.3%), China (1.6%), Japan (1.6%), Korea (1.3%), and New Zealand (1.3%). Some of the most discussed cases occurred in the USA; the Imanishi-Kari, Gallo, and Schön cases [9, 10]. Intensely discussed cases are also associated with Canada (Fisher/Poisson and Olivieri cases), the UK (Wakefield and CNEP trial cases), South Korea (Hwang case), and Japan (RIKEN case) [12, 14]. In terms of percentages, North America and Europe stand out in the number of articles (Fig. 7).

##### Case analysis

The case analysis involved the location where the case took place, taking into account the institutions involved in the case. For cases involving more than one country, all the countries were considered. Three cases were excluded from the analysis due to insufficient information. In the case analysis, 40 countries were involved in 235 different cases (Additional file 4). Our findings show that most of the reported cases occurred in the USA and the United Kingdom (59.6% and 9.8% of cases, respectively). In addition, a number of cases occurred in Canada (6.0%), Japan (5.5%), China (2.1%), and Germany (2.1%). In terms of percentages, North America and Europe stand out in the number of cases (Fig. 7). To enable comparison, we have additionally collected the number of published documents according to country distribution, available on SCImago Journal & Country Rank [16]. The numbers correspond to the documents published from 1996 to 2019. The USA occupies the first place in the number of documents, with 21.9%, followed by China (11.1%), UK (6.3%), Germany (5.5%), and Japan (4.9%).

#### Field of science

**Fig. 8:**
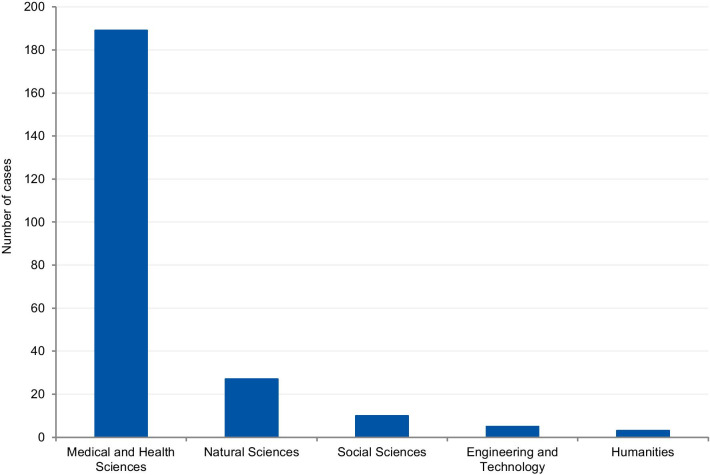
Field of science from the analysis of cases

The cases were classified according to the field of science. Four cases (1.7%) could not be classified due to insufficient information. Where information was available, 80.8% of cases were from the Medical and Health Sciences, 11.5% from the Natural Sciences, 4.3% from Social Sciences, 2.1% from Engineering and Technology, and 1.3% from Humanities (Fig. 8). Additionally, we have retrieved the number of published documents according to scientific field distribution, available on SCImago [16]. Of the total number of scientific publications, 41.5% are related to natural sciences, 22% to engineering, 25.1% to health and medical sciences, 7.8% to social sciences, 1.9% to agricultural sciences, and 1.7% to the humanities.

#### Sanctions

This variable aimed to collect information on possible consequences and sanctions imposed by funding agencies, scientific journals and/or institutions. 97 cases could not be classified due to insufficient information. 141 cases were included. Each case could potentially include more than one outcome. Most of cases (45.4%) involved paper retraction, followed by exclusion from funding applications (35.5%). (Table 2).

**Table 2:** Frequency of different sanctions in the analysis of cases

Sanction	Number of cases	Percentage (%)	Percentage of cases (n = 141) (%)
Paper retraction	64	33.3	45.4
Excluded from fund applications	50	26.0	35.5
Barred from service	22	11.5	15.6
Fired or suspended	18	9.4	12.8
Paper correction	12	6.3	8.5
Resignation	7	3.6	5.0
Trial	6	3.1	4.3
Manuscript rejection	5	2.6	3.5
Prison	3	1.6	2.1
Study halted	3	1.6	2.1
Fines / Restitution	2	1.0	1.4

## Discussion

RE and RI cases have been increasingly discussed publicly, affecting public attitudes towards scientists and raising awareness about ethical issues, violations, and their wider consequences [5]. Different approaches have been applied in order to quantify and address research misbehaviors [5, 17–19]. However, most cases are investigated confidentially and the findings remain undisclosed even after the investigation [19, 20]. Therefore, the study aimed to collect the RE and RI cases available in the scientific literature, understand how the cases are discussed, and identify the potential of case descriptions to raise awareness on RE and RI.

### Articles

We collected and analyzed 500 detailed case descriptions from 388 articles and our results show that they mostly relate to extensively discussed and notorious cases. Approximately half of all included cases was mentioned in at least two different articles, and the top ten most commonly mentioned cases were discussed in 132 articles.

The prominence of certain cases in the literature, based on the number of duplicated cases we found (e.g. Hwang case), can be explained by the type of article in which cases are discussed and the type of violation involved in the case. In the article genre analysis, 33% of the cases were described in the news section of scientific publications. Our findings show that almost all article genres discuss those cases that are new and in vogue. Once the case appears in the public domain, it is intensely discussed in the media and by scientists, and some prominent cases have been discussed for more than 20 years (Table 1). Misconduct and retraction notices were exceptions in the article genre analysis, as they presented mostly unique cases. The misconduct notices were mainly found on the NIH repository, which is indexed in the searched databases. Some federal funding agencies like NIH usually publicize investigation findings associated with the research they fund. The results derived from the NIH repository also explains the large proportion of articles from the US (61.9%). However, in some cases, only a few details are provided about the case. For cases that have not received federal funding and have not been reported to federal authorities, the investigation is conducted by local institutions. In such instances, the reporting of findings depends on each institution’s policy and willingness to disclose information [21]. The other exception involves retraction notices. Despite the existence of ethical guidelines [22], there is no uniform and a common approach to how a journal should report a retraction. The Retraction Watch website suggests two lists of information that should be included in a retraction notice to satisfy the minimum and optimum requirements [22, 23]. As well as disclosing the reason for the retraction and information regarding the retraction process, optimal notices should include: (I) the date when the journal was first alerted to potential problems; (II) details regarding institutional investigations and associated outcomes; (III) the effects on other papers published by the same authors; (IV) statements about more recent replications only if and when these have been validated by a third party; (V) details regarding the journal’s sanctions; and (VI) details regarding any lawsuits that have been filed regarding the case. The lack of transparency and information in retraction notices was also noted in studies that collected and evaluated retractions [24]. According to Resnik and Dinse [25], retractions notices related to cases of misconduct tend to avoid naming the specific violation involved in the case. This study found that only 32.8% of the notices identify the actual problem, such as fabrication, falsification, and plagiarism, and 58.8% reported the case as replication failure, loss of data, or error. Potential explanations for euphemisms and vague claims in retraction notices authored by editors could pertain to the possibility of legal actions from the authors, honest or self-reported errors, and lack of resources to conduct thorough investigations. In addition, the lack of transparency can also be explained by the conflicts of interests of the article’s author(s), since the notices are often written by the authors of the retracted article.

The analysis of violations/ethical issues shows the dominance of fabrication and falsification cases and explains the high prevalence of prominent cases. Non-adherence to laws and regulations (REC approval, informed consent, and data protection) was the second most prevalent issue, followed by patient safety, plagiarism, and conflicts of interest. The prevalence of the five most tagged violations in the case analysis was higher than the prevalence found in the analysis of articles that involved the same violations. The only exceptions are fabrication and falsification cases, which represented 45% of the tagged violations in the analysis of cases, and 59.1% in the article analysis. This disproportion shows a predilection for the publication of discussions related to fabrication and falsification when compared to other serious violations. Complex cases involving these types of violations make good headlines and this follows a custom pattern of writing about cases that catch the public and media’s attention [26]. The way cases of RE and RI violations are explored in the literature gives a sense that only a few scientists are “the bad apples” and they are usually discovered, investigated, and sanctioned accordingly. This implies that the integrity of science, in general, remains relatively untouched by these violations. However, studies on misconduct determinants show that scientific misconduct is a systemic problem, which involves not only individual factors, but structural and institutional factors as well, and that a combined effort is necessary to change this scenario [27, 28].

### Analysis of cases

#### Date

A notable increase in RE and RI cases occurred in the 1990s, with a gradual increase until approximately 2006. This result is in agreement with studies that evaluated paper retractions [24, 29]. Although our study did not focus only on retractions, the trend is similar. This increase in cases should not be attributed only to the increase in the number of publications, since studies that evaluated retractions show that the percentage of retraction due to fraud has increased almost ten times since 1975, compared to the total number of articles. Our results also show a gradual reduction in the number of cases from 2011 and a greater drop in 2015. However, this reduction should be considered cautiously because many investigations take years to complete and have their findings disclosed. ORI has shown that from 2001 to 2010 the investigation of their cases took an average of 20.48 months with a maximum investigation time of more than 9 years [24].

#### Geographical distribution

The countries from which most cases were reported were the USA (59.6%), the UK (9.8%), Canada (6.0%), Japan (5.5%), and China (2.1%). When analyzed by continent, the highest percentage of cases took place in North America, followed by Europe, Asia, Oceania, Latin America, and Africa. The predominance of cases from the USA is predictable, since the country publishes more scientific articles than any other country, with 21.8% of the total documents, according to SCImago [16]. However, the same interpretation does not apply to China, which occupies the second position in the ranking, with 11.2%. These differences in the geographical distribution were also found in a study that collected published research on research integrity [30]. The results found by Aubert Bonn and Pinxten (2019) show that studies in the United States accounted for more than half of the sample collected, and although China is one of the leaders in scientific publications, it represented only 0.7% of the sample. Our findings can also be explained by the search strategy that included only keywords in English. Since the majority of RE and RI cases are investigated and have their findings locally disclosed, the employment of English keywords and terms in the search strategy is a limitation. Moreover, our findings do not allow us to draw inferences regarding the incidence or prevalence of misconduct around the world. Instead, it shows where there is a culture of publicly disclosing information and openly discussing RE and RI cases in English documents.

#### Scientific field analysis

The results show that 80.8% of reported cases occurred in the medical and health sciences whilst only 1.3% occurred in the humanities. This disciplinary difference has also been observed in studies on research integrity climates. A study conducted by Haven and colleagues, [28] associated seven subscales of research climate with the disciplinary field. The subscales included: (1) Responsible Conduct of Research (RCR) resources, (2) regulatory quality, (3) integrity norms, (4) integrity socialization, (5) supervisor/supervisee relations, (6) (lack of) integrity inhibitors, and (7) expectations. The results, based on the seven subscale scores, show that researchers from the humanities and social sciences have the lowest perception of the RI climate. By contrast, the natural sciences expressed the highest perception of the RI climate, followed by the biomedical sciences. There are also significant differences in the depth and extent of the regulatory environments of different disciplines (e.g. the existence of laws, codes of conduct, policies, relevant ethics committees, or authorities). These findings corroborate our results, as those areas of science most familiar with RI tend to explore the subject further, and, consequently, are more likely to publish case details. Although the volume of published research in each research area also influences the number of cases, the predominance of medical and health sciences cases is not aligned with the trends regarding the volume of published research. According to SCImago Journal & Country Rank [16], natural sciences occupy the first place in the number of publications (41,5%), followed by the medical and health sciences (25,1%), engineering (22%), social sciences (7,8%), and the humanities (1,7%). Moreover, biomedical journals are overrepresented in the top scientific journals by IF ranking, and these journals usually have clear policies for research misconduct. High-impact journals are more likely to have higher visibility and scrutiny, and consequently, more likely to have been the subject of misconduct investigations. Additionally, the most well-known general medical journals, including NEJM, The Lancet, and the BMJ, employ journalists to write their news sections. Since these journals have the resources to produce extensive news sections, it is, therefore, more likely that medical cases will be discussed.

#### Violations analysis

In the analysis of violations, the cases were categorized into major and minor misbehaviors. Most cases involved data fabrication and falsification, followed by cases involving non-adherence to laws and regulations, patient safety, plagiarism, and conflicts of interest. When classified by categories, 12.5% of the tagged violations involved issues in the study design, 16.4% in data collection, 56.0% in reporting, and 15.1% involved collaboration issues. Approximately 80% of the tagged violations involved serious research misbehaviors, based on the ranking of research misbehaviors proposed by Bouter and colleagues. However, as demonstrated in a meta-analysis by Fanelli (2009), most self-declared cases involve questionable research practices. In the meta-analysis, 33.7% of scientists admitted questionable research practices, and 72% admitted when asked about the behavior of colleagues. This finding contrasts with an admission rate of 1.97% and 14.12% for cases involving fabrication, falsification, and plagiarism. However, Fanelli’s meta-analysis does not include data about research misbehaviors in its wider sense but focuses on behaviors that bias research results (i.e. fabrication and falsification, intentional non-publication of results, biased methodology, misleading reporting). In our study, the majority of cases involved FFP (66.4%). Overrepresentation of some types of violations, and underrepresentation of others, might lead to misguided efforts, as cases that receive intense publicity eventually influence policies relating to scientific misconduct and RI [20].

#### Sanctions analysis

The five most prevalent outcomes were paper retraction, followed by exclusion from funding applications, exclusion from service or position, dismissal and suspension, and paper correction. This result is similar to that found by Redman and Merz [31], who collected data from misconduct cases provided by the ORI. Moreover, their results show that fabrication and falsification cases are 8.8 times more likely than others to receive funding exclusions. Such cases also received, on average, 0.6 more sanctions per case. Punishments for misconduct remain under discussion, ranging from the criminalization of more serious forms of misconduct [32] to social punishments, such as those recently introduced by China [33]. The most common sanction identified by our analysis—paper retraction—is consistent with the most prevalent types of violation, that is, falsification and fabrication.

#### Publicizing scientific misconduct

The lack of publicly available summaries of misconduct investigations makes it difficult to share experiences and evaluate the effectiveness of policies and training programs. Publicizing scientific misconduct can have serious consequences and creates a stigma around those involved in the case. For instance, publicized allegations can damage the reputation of the accused even when they are later exonerated [21]. Thus, for published cases, it is the responsibility of the authors and editors to determine whether the name(s) of those involved should be disclosed. On the one hand, it is envisaged that disclosing the name(s) of those involved will encourage others in the community to foster good standards. On the other hand, it is suggested that someone who has made a mistake should have the right to a chance to defend his/her reputation. Regardless of whether a person's name is left out or disclosed, case reports have an important educational function and can help guide RE- and RI-related policies [34]. A recent paper published by Gunsalus [35] proposes a three-part approach to strengthen transparency in misconduct investigations. The first part consists of a checklist [36]. The second suggests that an external peer reviewer should be involved in investigative reporting. The third part calls for the publication of the peer reviewer’s findings.

#### Limitations

One of the possible limitations of our study may be our search strategy. Although we have conducted pilot searches and sensitivity tests to reach the most feasible and precise search strategy, we cannot exclude the possibility of having missed important cases. Furthermore, the use of English keywords was another limitation of our search. Since most investigations are performed locally and published in local repositories, our search only allowed us to access cases from English-speaking countries or discussed in academic publications written in English. Additionally, it is important to note that the published cases are not representative of all instances of misconduct, since most of them are never discovered, and when discovered, not all are fully investigated or have their findings published. It is also important to note that the lack of information from the extracted case descriptions is a limitation that affects the interpretation of our results. In our review, only 25 retraction notices contained sufficient information that allowed us to include them in our analysis in conformance with the inclusion criteria. Although our search strategy was not focused specifically on retraction and misconduct notices, we believe that if sufficiently detailed information was available in such notices, the search strategy would have identified them.

## Conclusion

Case descriptions found in academic journals are dominated by discussions regarding prominent cases and are mainly published in the news section of journals. Our results show that there is an overrepresentation of biomedical research cases over other scientific fields when compared with the volume of publications produced by each field. Moreover, published cases mostly involve fabrication, falsification, and patient safety issues. This finding could have a significant impact on the academic representation of ethical issues for RE and RI. The predominance of fabrication and falsification cases might diverge the attention of the academic community from relevant but less visible violations and ethical issues, and recently emerging forms of misbehaviors.

## Supplementary Information

**Additional file 1**. Pilot search and search strategy.

**Additional file 2**. List of Major and minor misbehavior items (Developed by Bouter LM, Tijdink J, Axelsen N, Martinson BC, ter Riet G. Ranking major and minor research misbehaviors: results from a survey among participants of four World Conferences on Research Integrity. Research integrity and peer review. 2016;1(1):17. https://doi.org/10.1186/s41073-016-0024-5).

**Additional file 3**. Table containing the number and percentage of countries included in the analysis of articles.

**Additional file 4**. Table containing the number and percentage of countries included in the analysis of the cases.

## Data Availability

This review has been developed by members of the EnTIRE project in order to generate information on the cases that will be made available on the Embassy of Good Science platform (www.embassy.science). The dataset supporting the conclusions of this article is available in the Open Science Framework (OSF) repository in https://osf.io/3xatj/?view_only=313a0477ab554b7489ee52d3046398b9.
