# Supplementary material for: A scoping review of the literature featuring research ethics and research integrity cases
Source: BMC Med Ethics. 2021 Apr 30;22:50. doi: 10.1186/s12910-021-00620-8 (PMC8086087; doi:10.1186/s12910-021-00620-8)
Supplement: Supplementary file 1 — Additional file 1. Pilot search and search strategy. [file 12910_2021_620_MOESM1_ESM.docx]

**A scoping review of the literature featuring research ethics and research integrity cases**
A.C.V Armond, B. Gordijn, J. Lewis, M. Hosseini, J. K. Bodnár, S. Holm, P. Kakuk

**Additional file 1**

**Pilot search and search strategy**

We conducted different pilot searches to reach the most feasible and precise search strategy using keywords and Boolean operators. At the piloting phase, we tested the search-term combinations suggested in the original protocol (available at https://ec.europa.eu/research/participants/documents/downloadPublic?documentIds=080166e5bde92120&appId=PPGMS) (“research ethics” and “case”) and (“research integrity” and “case”) using PubMed and Science Direct. Two researchers independently checked the results and screened the content (titles, journal names, genres, abstracts) of the first hundred results. However, this search strategy resulted in a huge number of results that could not be adequately accounted for within the limits of the project due to a lack of time and resources. The term “case” or “case study” is broad, and its inclusion (using the Boolean operator “AND”) both excluded important results and included many results unrelated to the topic of RE+RI. For example, using (“research ethics” AND “case”) as a keyword set in SCOPUS, PubMed, Science Direct, Web of Science, JSTOR, and Ovid, 57,954 results were retrieved. As a team, we discussed the feasibility and precision of the keywords suggested in the protocol. Subsequently, we tested different keywords in order to generate manageable results. We decided to continue to refine keywords by testing candidate keywords and their variations in SCOPUS, PubMed, Science Direct, Web of Science, JSTOR, and Ovid. We also tested two hundred results for sensitivity. If the initial two hundred results included a high number of irrelevant articles, then we had suitable justification to modify the search terms. To identify research ethics (RE) cases in the academic literature, we developed the search term ((“research ethics”) AND (“violation” OR “unethical” OR “misconduct”)), and ((“research integrity”) AND (“violation” OR “unethical” OR “misconduct”)). It generated a manageable number of precise and relevant results.
