# Supplementary material for: A scoping review of the literature featuring research ethics and research integrity cases
Source: BMC Med Ethics. 2021 Apr 30;22:50. doi: 10.1186/s12910-021-00620-8 (PMC8086087; doi:10.1186/s12910-021-00620-8)
Supplement: Supplementary file 3 — Additional file 3. Table containing the number and percentage of countries included in the analysis of articles. [file 12910_2021_620_MOESM3_ESM.docx]

| **Origin of articles** | **Frequency** | **Percentage** | **Valid percentage** |
| --- | --- | --- | --- |
| Missing information | 81 | 20.88 | - |
| Australia | 10 | 2.58 | 3.26 |
| Brazil | 1 | 0.26 | 0.33 |
| Canada | 15 | 3.87 | 4.89 |
| China | 5 | 1.29 | 1.63 |
| Denmark | 2 | 0.52 | 0.65 |
| France | 1 | 0.26 | 0.33 |
| Hungary | 1 | 0.26 | 0.33 |
| India | 3 | 0.77 | 0.98 |
| Iran | 2 | 0.52 | 0.65 |
| Ireland | 1 | 0.26 | 0.33 |
| Japan | 5 | 1.29 | 1.63 |
| Korea | 4 | 1.03 | 1.30 |
| Mexico | 1 | 0.26 | 0.33 |
| Netherlands | 2 | 0.52 | 0.65 |
| New Zealand | 4 | 1.03 | 1.30 |
| Norway | 2 | 0.52 | 0.65 |
| Portugal | 1 | 0.26 | 0.33 |
| Singapore | 1 | 0.26 | 0.33 |
| South Africa | 2 | 0.52 | 0.65 |
| Spain | 2 | 0.52 | 0.65 |
| Sweden | 2 | 0.52 | 0.65 |
| Switzerland | 3 | 0.77 | 0.98 |
| Taiwan | 1 | 0.26 | 0.33 |
| Turkey | 2 | 0.52 | 0.65 |
| UK | 44 | 11.34 | 14.33 |
| USA | 190 | 48.97 | 61.89 |
|  | 388 | 100.00 | 100.00 |
