# Supplementary material for: A scoping review of the literature featuring research ethics and research integrity cases
Source: BMC Med Ethics. 2021 Apr 30;22:50. doi: 10.1186/s12910-021-00620-8 (PMC8086087; doi:10.1186/s12910-021-00620-8)
Supplement: Supplementary file 4 — Additional file 4. Table containing the number and percentage of countries included in the analysis of the cases. [file 12910_2021_620_MOESM4_ESM.docx]

| **Countries of cases** | **Number of cases** | **Percentage** | **Percentage of cases (n=235)** |
| --- | --- | --- | --- |
| USA | 140 | 55.12 | 59.6 |
| UK | 23 | 9.06 | 9.8 |
| Canada | 14 | 5.51 | 6.0 |
| Japan | 13 | 5.12 | 5.5 |
| China | 5 | 1.97 | 2.1 |
| Germany | 5 | 1.97 | 2.1 |
| Netherlands | 4 | 1.57 | 1.7 |
| France | 3 | 1.18 | 1.3 |
| Romania | 3 | 1.18 | 1.3 |
| Sweden | 3 | 1.18 | 1.3 |
| Australia | 2 | 0.79 | 0.9 |
| Austria | 2 | 0.79 | 0.9 |
| Croatia | 2 | 0.79 | 0.9 |
| Denmark | 2 | 0.79 | 0.9 |
| Guatemala | 2 | 0.79 | 0.9 |
| India | 2 | 0.79 | 0.9 |
| New Zealand | 2 | 0.79 | 0.9 |
| South Africa | 2 | 0.79 | 0.9 |
| Turkey | 2 | 0.79 | 0.9 |
| Uganda | 2 | 0.79 | 0.9 |
| Brazil | 1 | 0.39 | 0.4 |
| Burkina Faso | 1 | 0.39 | 0.4 |
| Congo | 1 | 0.39 | 0.4 |
| Dominican Republic | 1 | 0.39 | 0.4 |
| Ethiopia | 1 | 0.39 | 0.4 |
| Iran | 1 | 0.39 | 0.4 |
| Ivory Coast | 1 | 0.39 | 0.4 |
| Italy | 1 | 0.39 | 0.4 |
| Kenya | 1 | 0.39 | 0.4 |
| Malawi | 1 | 0.39 | 0.4 |
| Nigeria | 1 | 0.39 | 0.4 |
| Norway | 1 | 0.39 | 0.4 |
| Poland | 1 | 0.39 | 0.4 |
| Scotland | 1 | 0.39 | 0.4 |
| Singapore | 1 | 0.39 | 0.4 |
| South Korea | 2 | 0.79 | 0.9 |
| Switzerland | 1 | 0.39 | 0.4 |
| Tanzania | 1 | 0.39 | 0.4 |
| Thailand | 1 | 0.39 | 0.4 |
| Zimbabwe | 1 | 0.39 | 0.4 |
